# Construction of a nomogram model for predicting vaginal birth after induction of labor in pregnant women with fetal growth restriction at term

**DOI:** 10.3389/fmed.2026.1650365

**Published:** 2026-01-28

**Authors:** Liping Huang, Ting Yao, Lin Lin, Wenqiang You, Mingxing Yan

**Affiliations:** 1 Fujian Maternity and Child Health Hospital, College of Clinical Medicine for Obstetrics & Gynecology and Pediatrics, Fuzhou, China; 2 College of Clinical Medicine for Obstetrics & Gynecology and Pediatrics, Fujian Medical University, Fuzhou, China

**Keywords:** Cook’s double balloon, dinoprostone, fetal growth restriction, induction of labor, nomogram

## Abstract

**Objectives:**

The study aimed to develop and validate a predictive nomogram model of vaginal delivery in women with fetal growth restriction (FGR) who undergo labor induction.

**Methods:**

A retrospective cohort study was conducted at Fujian Maternity and Child Health Hospital, involving 507 singleton pregnancies complicated with fetal growth restriction (FGR) between October 2017 and December 2022. These pregnancies underwent labor induction with dinoprostone or Cook’s double balloon. The cohort was randomly divided into two groups: 75% of pregnancies (*n* = 380) were utilized to identify independent factors associated with vaginal delivery using multi-logistic regression and develop a predictive nomogram model, while 25% (*n* = 127) were allocated for internal validation of the model.

**Results:**

A predictive nomogram model was constructed with five validated factors including maternal age, multiparity, oligohydramnios (borderline significant), Bishop score after cervical ripening and Cook’s double balloon. The trained and validated area under the curve were 0.811 (95% confident interval 0.757–0.865) and 0.760 (95% confident interval 0.669–0.860), respectively. The Hosmer-Lemeshow test indicated no statistically significant difference between the predicted and observed outcomes (*P* > 0.050). The clinical decision curve demonstrated that both the model and the validation groups achieved the greatest net benefit at threshold probability values ranging from 0.20 to 0.95 and exceeding 0.40, respectively.

**Conclusion:**

The nomogram model could be utilized to inform patients with FGR about their success of vaginal delivery during labor induction. Moreover, this model established a foundation for clinical intervention and the development of personalized medical treatment strategies.

## 1 Introduction

Fetal growth restriction (FGR) is a condition in which the fetus does not achieve its biological growth potential, affecting approximately 10% of cases (1). FGR is a significant contributor to infant morbidity and mortality, ranking second only to premature birth. Furthermore, FGR has been associated with long-term complications including impaired neurodevelopment and cardiovascular disease(s) in adulthood (2). Estimated fetal weight (EFW) was calculated using the Hadlock IV formula (3), and FGR was defined as sonographic EFW < 10th percentile for gestational age per the 2013 World Health Organization (WHO) fetal growth curves (4). Chronic fetal hypoxia and undernutrition during human pregnancy may cause FGR. Given the increased risk for stillbirth in fetuses with FGR beyond 37 weeks, it is recommended by national and international guidelines (5) that such pregnancies be induced at 37–39 weeks’ gestation. However, these guidelines do not provide specific recommendations for the optimal method of labor induction to ensure vaginal delivery in pregnancies affected by FGR. Villalaín et al. (6) suggested that the Foley balloon may be a preferable approach due to its lower incidence of uterine tachysystole compared with prostaglandins. In contrast, Duro-Gomez et al. (7) reported that dinoprostone exhibited greater efficacy than that of the Cook’s double balloon in induction of FGR pregnancies. Furthermore, some studies (8, 9) have identified additional factors that contribute to vaginal delivery in FGR. Hence, identifying factors that contribute to vaginal delivery during labor induction holds significant importance. Additionally, there is a necessity to establish a universally applicable predictive model to evaluate the probability of vaginal delivery in pregnancies with FGR.

The objective of this retrospective cohort study was to examine the variables associated with efficacious labor induction in pregnancies with FGR, and to develop and internally validate a clinical predictive model for vaginal delivery following induction.

## 2 Materials and methods

### 2.1 Study population

A retrospective cohort study was undertaken at Fujian Maternity and Child Health Hospital, including a consecutive cohort of pregnancies with FGR between October 2017 and December 2022. Data were collected from electronic medical records. This study was approved by ethics approval from Ethics Committee of Fujian Maternity and Child Health Hospital (Approval number: 2023KY016). Given the retrospective design of the study and the use of anonymized data extracted from existing electronic medical records (with no additional interventions or risks to participants), the Ethics Committee waived the requirement for research-specific informed consent.

**FIGURE 1:**
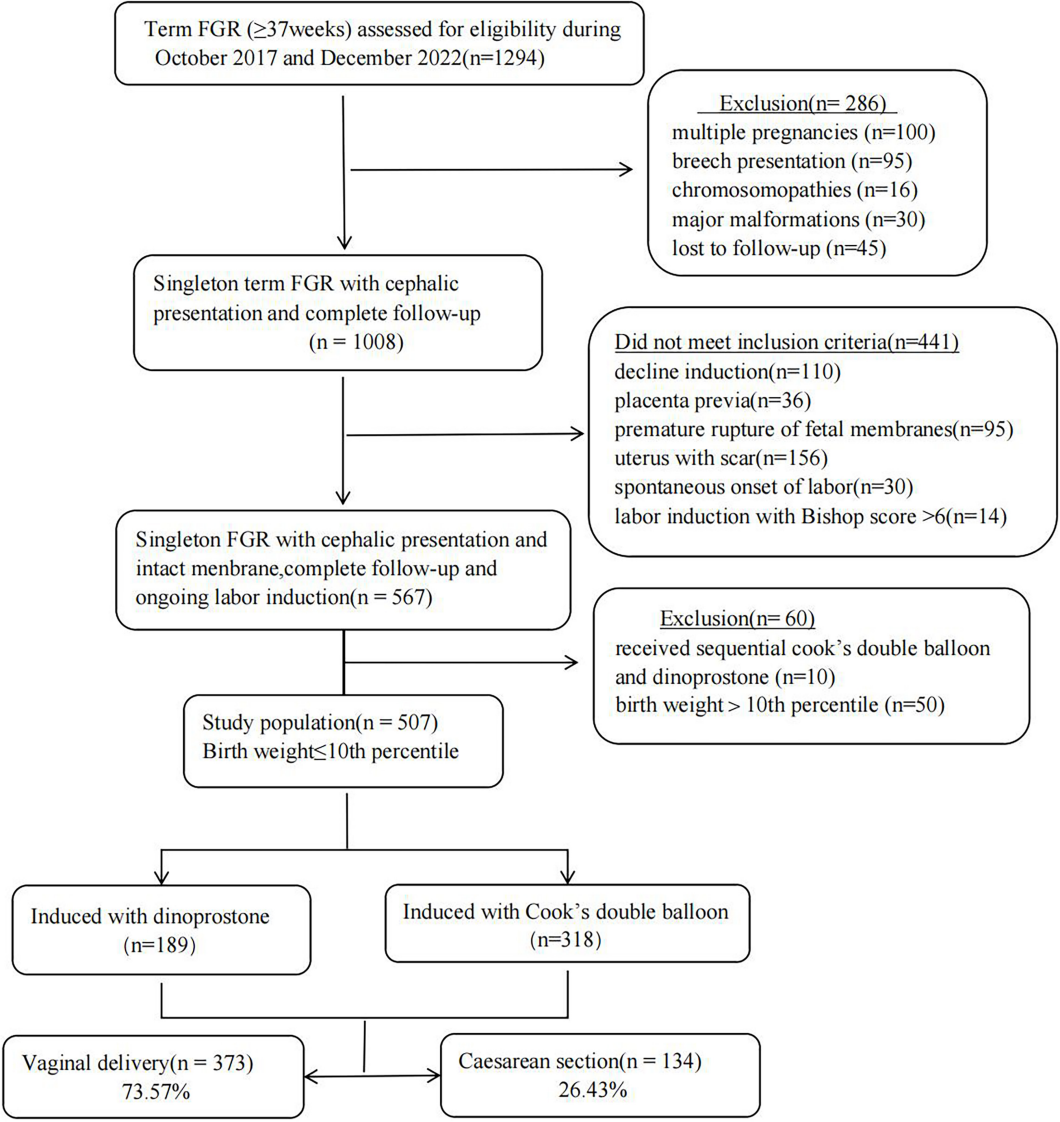
Flowchart of the enrolled patients.

The inclusion criteria was singleton pregnancy diagnosed with FGR at full-term. Gestational age was determined by last menstrual period (LMP) and confirmed by first-trimester crown-rump length (CRL) ultrasound (per ISUOG guidelines) (10). Multiple pregnancies, infants with congenital malformations, fetal chromosomal abnormalities, premature rupture of membranes and a history of uterine scar (previous cesarean section, uterine surgery involving myometrial incision, or other procedures causing permanent uterine wall scarring), as well as those lost to follow-up were excluded. A total of 567 cases involving cephalic presentation with cervical Bishop score < 6 points on the first assessment performed within 24 h before labor induction underwent the induction process. From this pool, cases of FGR diagnosed before delivery and had a birth weight <10th percentile were specifically selected resulting in a sample size of 507. The selection process is illustrated in Figure 1. Subsequently, the final FGR cohort was randomly divided into two groups: one (*n* = 380) for constructing the prediction model, and the other (*n* = 127) for internal validation of the final model.

### 2.2 Treatment protocol for FGR and induction methods

As per department protocol, FGR patients were subjected to routine ultrasound and fetal heart rate monitoring before labor induction. The same senior physician assessed cervical status using the Bishop score. Additionally, FGR patients were provided with information regarding the procedures and the potential risks associated with labor induction, such as the use of Cook’s double balloon and dinoprostone. Written clinical informed consent was obtained from all patients to document their voluntary choice of cervical ripening method, in compliance with standard clinical ethical practices. In accordance with manufacturer’s instructions, a 10 mg dose of dinoprostone was inserted into the posterior vaginal fornix. The Cook’s double balloon was introduced by injecting 80 ml of saline solution into both the intrauterine and intravaginal balloons. Ultimately, 189 patients were induced using dinoprostone and 318 using Cook’s double balloon.

According to our labor induction guidelines, both approaches were maintained for a maximum duration of 12 h with continuous cardiotocographic monitoring. If patients entered regular labor progression (defined as cervical dilation ≥ 2 cm with regular uterine contractions every 3 min, lasting 40–60 s each),experienced membrane rupture, had non-reassuring fetal heart rate, encountered placental abruption, or had uterine tachysystole (>5 uterine contractions in 10 min), the device (either balloon or dinoprostone) was removed. If labor had not progressed to regular labor progression after 12 h of device placement, they were to be removed. Subsequently, intravenous oxytocin was initiated at a rate of 2.5 mIU/min and adjusted every 15 min according to uterine contractions. Induction failure was defined as the inability to achieve cervical dilation ≥ 2 cm within 12 h of cervical ripening device placement (Cook’s double balloon or dinoprostone) despite adequate uterine contractions (≥ 3 contractions in 10 min, lasting 40–60 s each) and 24 h of oxytocin augmentation. CS was recommended in cases of failed labor induction.

### 2.3 Data collection

The primary variable was the mode of childbirth, with vaginal delivery defined as successful induction. Maternal characteristics including age, height, weight, pre-pregnancy body mass index (BMI), BMI at delivery, parity, as well as gestational age at admission and delivery, maternal complications such as gestational diabetes and pregnancy-induced hypertension, EFW, induction details such as Bishop scores at admission and after induction, induction methods, mode of delivery, perinatal events, intrapartum complications, and neonatal birth weight, Apgar scores at 1 min and 5 min, and admission to the neonatal intensive care unit (NICU) were all collected.

### 2.4 Statistical analysis

Qualitative variables are expressed as frequency and percentage, whereas quantitative variables are expressed as mean ± standard deviation (SD) or median and interquartile range. Pearson χ2 test or Fisher’s exact test were used to compare qualitative variables, while continuous variables were assessed using Student’s *t*-test or Kruskal-Wallis rank test.

Logistic regression was used to construct a predictive model for successful vaginal delivery in patients with FGR undergoing induction. The original dataset was randomly divided into 75% (training set) and 25% (validation set). For variable selection in the multivariate model: (1) All variables with a two-sided *P*-value < 0.10 in univariate logistic regression were initially included as candidate predictors; (2) A stepwise backward selection method was then applied, with variables retained based on the minimum value of the Akaike Information Criterion (AIC) to balance model fit and parsimony; (3) Clinical relevance of retained variables was further validated against existing literature and expert consensus to ensure biological plausibility. This model estimated odds ratio (OR) and corresponding 95% confidence interval (CI) to summarize the associations. Additionally, a graphic nomogram was generated to visually represent the logistic regression model.

Evaluation of model discrimination was performed by calculating the area under the curve (AUC) of the receiver operating characteristic (ROC) curve. The regression model was plotted as a ROC curve, and the AUC along with its corresponding 95% CI are reported with an AUC >0.7 indicating exceptional discriminatory capacity. Calibration of the nomogram model was evaluated using the Hosmer-Lemeshow test (*p* < 0.050 indicated a significant lack of fit) and a calibration curve. The calibration plot of the nomogram model exhibited the predicted and actual probabilities for each patient, with a correlation close to the ideal 45° line, indicating a strong association. Decision curve analysis (DCA) was performed to evaluate the clinical utility of the nomogram. Statistical analysis was performed using R version 4.3.0 (R Project for Statistical Computing, Vienna, Austria) and SPSS version 24.0 (IBM Corp, Armonk, NY, United States).

## 3 Results

### 3.1 Cohort characteristics

A cohort of 507 pregnant women with diagnosed FGR was ultimately included after excluding 60 patients, including 50 whose neonatal birth weights > 10th percentile and 10 patients who received both Cook’s double balloon and dinoprostone in induction of labor (Figure 1). Among this cohort, 134 underwent CS (26.43%) and 373 had vaginal deliveries (73.57%). The training set comprised 380 (75%) subjects, whereas the validation set comprised 127(25%). Demographic and obstetric characteristics are summarized in Table 1. There were no statistically significant differences (*P* > 0.050) between the training and validation set, supporting the reliability of the random allocation.

**TABLE 1:** Demographic and clinical characteristics of patients in training and validation set.

Variable	Total (*n* = 507)	Train set (*n* = 380)	Valid set (*n* = 127)	Statistic	*P*
**Antenatal variables**					
Maternal age, years	28.59 ± 4.36	28.46 ± 4.43	28.95 ± 4.16	t = −1.095	0.274
Gravidity	1.62 ± 0.95	1.65 ± 1.01	1.53 ± 0.73	t = 1.260	0.208
Previous vaginal delivery *n* (%)				χ^2^ = 0.533	0.766
0	383 (75.54)	290 (76.32)	93 (73.23)	–	–
1	112 (22.09)	81 (21.32)	31 (24.41)	–	–
≥2	12 (2.37)	9 (2.37)	3 (2.36)	–	–
Height, cm	158.95 ± 5.16	158.92 ± 5.20	159.04 ± 5.06	t = −0.238	0.812
Pre-pregnancy weight (kg)	50.44 ± 7.34	50.25 ± 7.58	51.00 ± 6.53	t = −1.002	0.317
Pre-pregnancy BMI_group				–	0.157
<18.5	154 (30.37)	123 (32.37)	31 (24.41)	–	–
18.5–24.9	312 (61.54)	224 (58.95)	88 (69.29)	–	–
25–29.9	38 (7.5)	31 (8.16)	7 (5.51)	–	–
≥30	3 (0.59)	2 (0.53)	1 (0.79)	–	–
Weight on admission (kg)	61.22 ± 9.31	60.86 ± 9.43	62.30 ± 8.90	t = −1.515	0.130
BMI at delivery_group				χ^2^ = 1.408	0.704
<18.5	27 (5.33)	21 (5.53)	6 (4.72)	–	–
18.5–24.9	210 (41.42)	161 (42.37)	49 (38.58)	–	–
25–29.9	205 (40.43)	148 (38.95)	57 (44.88)	–	–
≥30	65 (12.82)	50 (13.16)	15 (11.81)	–	–
Gestational age at admission				χ^2^ = 0.053	0.818
37–39	252 (49.7)	190 (50.00)	62 (48.82)	–	–
>39	255 (50.3)	190 (50.00)	65 (51.18)	–	–
Bishop score on admission	2.85 ± 0.86	2.84 ± 0.88	2.85 ± 0.79	t = −0.064	0.949
Gestational diabetes, *n* (%)	74 (14.6)	58 (15.26)	16 (12.60)	χ^2^ = 0.542	0.462
Gestational hypertension, *n* (%)	34 (6.71)	30 (7.89)	4 (3.15)	χ^2^ = 3.426	0.064
Oligoamnios, *n* (%)	59 (11.64)	43 (11.32)	16 (12.60)	χ^2^ = 0.152	0.696
Estimated fetal weight, g	2650.20 ± 266.55	2655.73 ± 268.05	2633.65 ± 262.36	t = 0.808	0.419
Intrapartum variables				–	–
Induction of labor, *n* (%)				χ^2^ = 0.247	0.619
Dinoprostone	189 (37.28)	144 (37.89)	45 (35.43)	–	–
Cook’s double balloon	318 (62.72)	236 (62.11)	82 (64.57)	–	–
Bishop score after cervical ripening	6.82 ± 1.60	6.80 ± 1.61	6.89 ± 1.56	t = −0.563	0.574
Oxytocin augmentation, *n* (%)	230 (45.36)	169 (44.47)	61 (48.03)	χ^2^ = 0.486	0.486
Intrapartum blood loss (ml)	262.45 ± 165.13	264.98 ± 156.98	254.91 ± 187.90	t = 0.595	0.552
Intrapartum pyrexia, *n* (%)	12 (2.37)	7 (1.84)	5 (3.94)	χ^2^ = 1.015	0.314
Variables at birth				–	–
Gestational age at birth_group				χ^2^ = 0.588	0.443
37–39	325 (64.1)	240 (63.16)	85 (66.93)	–	–
>39	182 (35.9)	140 (36.84)	42 (33.07)	–	–
Neonatal birth weight, g	2595.37 ± 206.66	2595.73 ± 211.26	2594.29 ± 193.02	t = 0.068	0.946
Neonatal birth weight < 3rd percentile, *n* (%)	89 (17.55)	64 (16.84)	25 (19.69)	χ^2^ = 0.532	0.466
APGAR at 1 min	9.89 ± 0.65	9.88 ± 0.65	9.91 ± 0.65	t = −0.321	0.748
APGAR < 7 at 5 min	3 (0.59)	2 (0.53)	1 (0.79)	–	>0.999
NICU, *n* (%)	18(3.55)	14 (3.68)	4 (3.15)	–	>0.999

BMI, body mass index; NICU, neonatal intensive care unit. Pearson χ^2^ test for qualitative variables with expected cell frequencies ≥ 5; Fisher’s exact test for qualitative variables with expected cell frequencies < 5 (APGAR < 7 at 5 min, NICU admission).

### 3.2 Nomogram predicting the success of vaginal delivery on labor induction construct and performance assessment

In the training cohort, univariate analysis demonstrated significant associations between various factors and successful vaginal birth in FGR patients. These factors included maternal age, maternal weight on admission, maternal pre-pregnancy BMI, parity, oligohydramnios, cervical Bishop score after device-induced ripening and use of Cook’s double balloon (*P* < 0.100) (Table 2). Further analysis revealed that maternal age (*P* < 0.001), multiparity (*P* = 0.040), Bishop score after cervical ripening (*P* < 0.001) and use of Cook’s double balloon (*P* = 0.004) were identified as significant independent predictors for vaginal delivery in FGR patients undergoing induction through subsequent multivariate regression analysis using AIC. Oligohydramnios exhibited a borderline significant association (*P* = 0.061) and was retained in the model due to its clinical relevance and contribution to model fit (Table 2).

**TABLE 2:** Univariate and multivariate logistic regression analysis of the predictors of success vaginal delivery in the training set.

Variables	Univariate analysis		Multivariate analysis	
	OR (95% CI)	*P*	aOR (95% CI)	a*P*
Maternal age, y	0.89 (0.85–0.94)	<0.001	0.88 (0.83–0.94)	<0.001
Gravidity	1.00 (0.79–1.25)	0.968	–	–
**Previous vaginal delivery (parity)**				
0	1.00 (reference)	–	1.00 (reference)	–
1	1.18 (0.66–2.10)	0.574	2.11(1.03–4.31)	0.040
2	0.29 (0.08–1.10)	0.070	0.32(0.08–1.34)	0.120
**Gestational age at admission**				
37–39	1.00 (reference)	–	–	–
>39	1.09 (0.55–2.15)	0.815	–	–
Height	1.02 (0.97–1.06)	0.476	–	–
Pre-pregnancy weight	0.98 (0.95–1.01)	0.129	–	–
**Pre-pregnancy BMI**				
<18.5	1.60 (0.95–2.71)	0.079	–	–
18.5–24.9	1.00 (reference)	–	–	–
25–29.9	0.86 (0.38–1.92)	0.711	–	–
≥30	0.41 (0.03–6.63)	0.529	–	–
Weight on admission	0.98 (0.95–1.00)	0.059	–	–
**BMI at delivery**				
<18.5	1.31 (0.63–2.74)	0.469	–	–
18.5–24.9	1.00 (reference)	–	–	–
25–29.9	0.65 (0.32–1.33)	0.236	–	–
≥30	3.34 (0.68–16.33)	0.137	–	–
**Gestational diabetes**				
No	1.00 (reference)	–	–	–
Yes	1.16 (0.61–2.23)	0.648	–	–
**Gestational hypertension**				
No	1.00 (reference)	–	–	–
Yes	1.21 (0.50–2.90)	0.675	–	–
**Oligoamnios**				
No	1.00 (reference)	–	1.00 (reference)	–
Yes	1.76 (0.90–3.42)	0.097	2.07(0.97–4.42)	0.061
Bishop score on admission	1.16 (0.89–1.51)	0.273	–	–
Estimated fetal weight (per 100 g increase)	1.03 (0.99–1.07)	0.194	–	–
**Gestational age at delivery**				
37–39	1.00 (reference)	–	–	–
≥39	1.08 (0.53–2.19)	0.834	–	–
Bishop score after cervical ripening	1.42 (1.21–1.66)	<0.001	1.48 (1.25–1.75)	<0.001
**Oxytocin augment**				
No	1.00 (reference)	–	–	–
Yes	0.94 (0.60–1.49)	0.800	–	–
**PROM during labor**				
No	1.00 (reference)	–	–	–
Yes	2.12 (0.86–5.23)	0.101	–	–
**Method of inducing labor**				
Dinoprostone	1.00 (reference)	–	1.00 (reference)	–
Cook’s double balloon	1.93 (1.22–3.06)	0.005	2.13(1.27–3.60)	0.004

BMI, body mass index; PROM, premature rupture of membranes.

**FIGURE 2:**
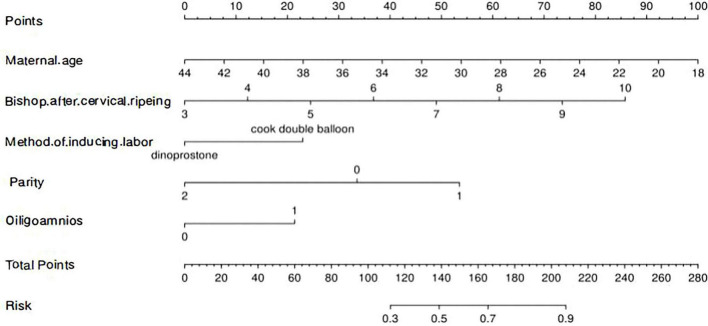
A nomogram for predicting success rate of vaginal delivery in labor induction for patients complicated with fetal growth restriction (FGR). The nomogram summed the points identified on the scale for each variable. The total points projected on the bottom scales indicate the probabilities of vaginal delivery in labor induction in FGR patients.

**FIGURE 3:**
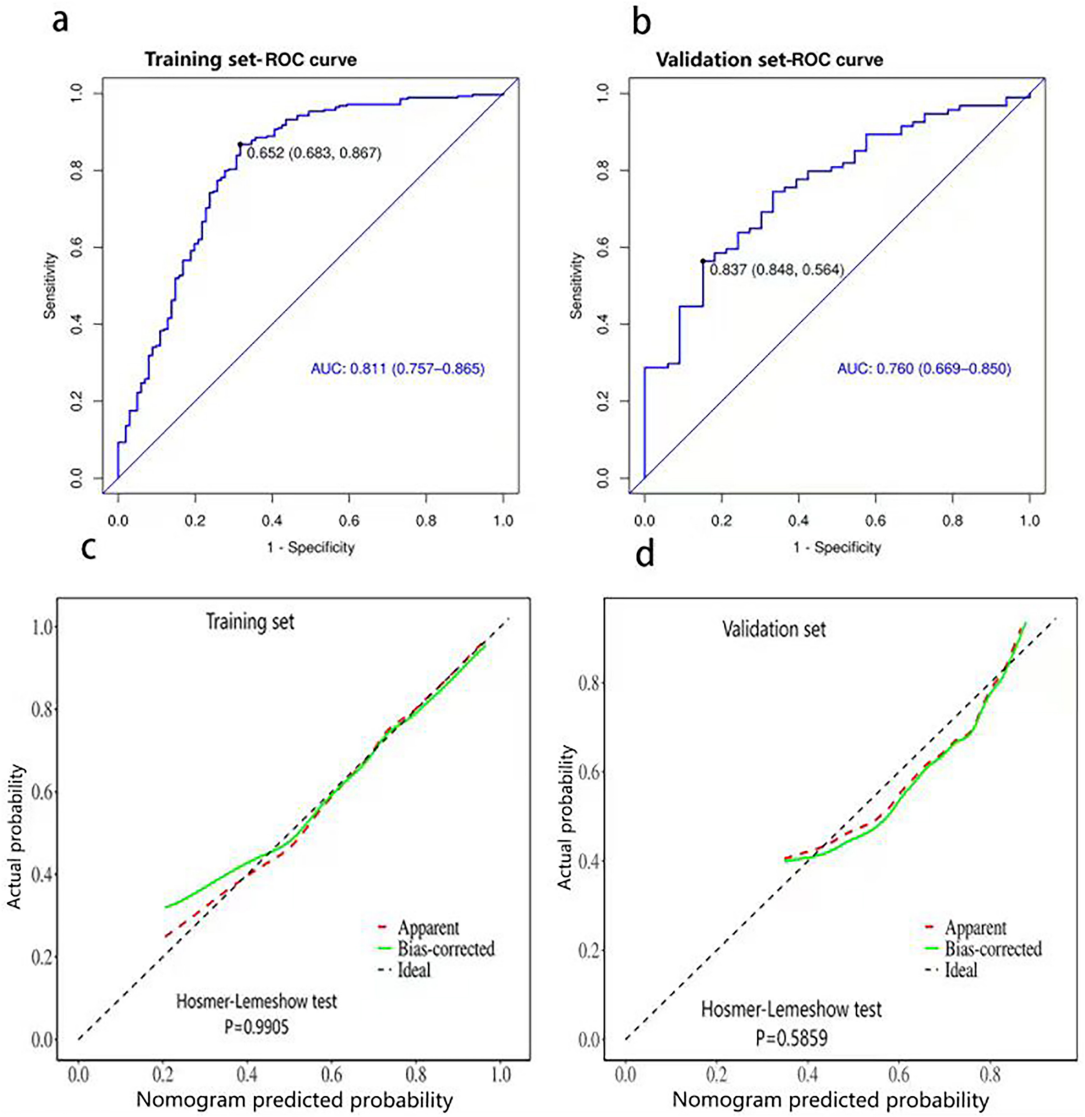
Discrimination and calibration of the nomogram for predicting success rate of vaginal delivery in induction of labor for patients complicated with fetal growth restriction (FGR). Receiver operator curve of the nomogram in the training set **(a)** and validation set **(b)**. Calibration curves of the nomogram in the training set **(c)** and validation set **(d)**. For the calibration curve, the X-axis indicates the probability of the nomogram prediction, and the Y-axis represents the actual operation probability. The Apparent red dashed line represents the entire cohort, the ideal black dashed line represents the corresponding perfect prediction, and the Bias-corrected green solid line represents the bias-corrected prediction by Bootstrapping (1,000 replicates), which indicates the observed model performance. The green solid line has a closer fit to the black dashed line, which indicates a better prediction. AUC, area under the curve; CI, confidence interval.

Subsequently, a nomogram was developed to predict the likelihood of successful vaginal delivery by incorporating these five predictors (Figure 2). The upper portion of the diagram features a point axis for assigning scores to each variable, with the cumulative score being calculated. Using this score, the probability of achieving successful labor induction can be anticipated. In the training dataset, the nomogram demonstrated favorable discriminatory ability, with an AUC of 0.811 (95% CI 0.757–0.865) (Figure 3a). The Youden index determined the optimal threshold value for diagnosis to be 0.652. Sensitivity and specificity were 68.30% and 86.70%, respectively. The calibration curve, which was adjusted using 1,000 bootstrap-resamples, exhibited strong concordance between the model’s predictions and actual observations in the training set (Figure 3c). Furthermore, the Hosmer-Lemeshow test yielded a non-significant *P*-value of 0.990, indicating exceptional calibration capability.

**FIGURE 4:**
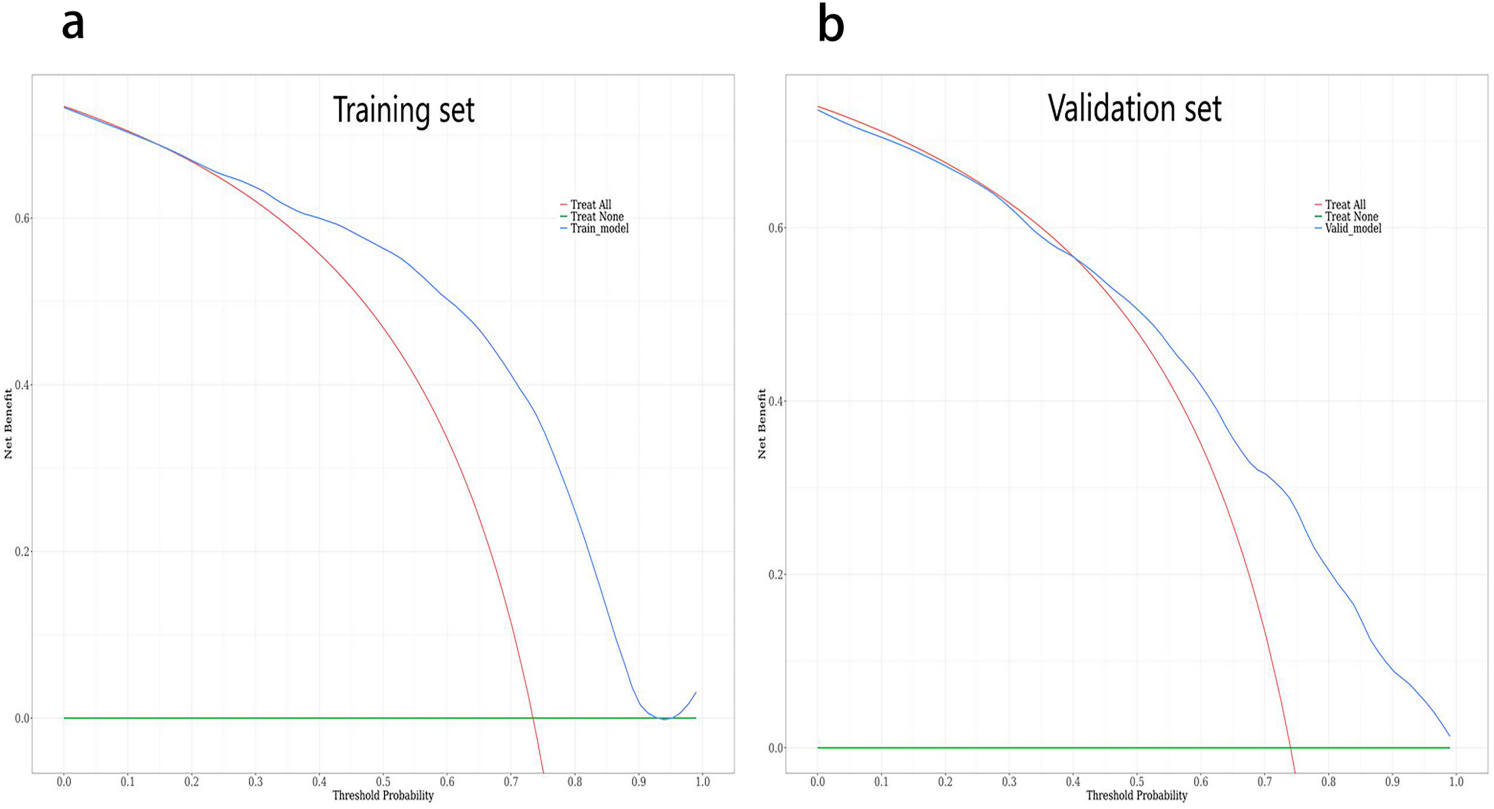
Decision curve analyses (DCA) depicting the clinical net benefit of the nomogram. **(a)** The decision curve analysis for the nomogram in the training set. **(b)** The decision curve analysis for the nomogram in the validation set. The x-axis shows the threshold probability. Threshold probability was defined as the minimum probability of disease at which further intervention would be warranted. The y-axis represents the net benefit, which is calculated across a range of threshold probabilities. The horizontal solid green line represents the assumption that no FGR patients with successful vaginal delivery after induction of labor were involved, and the solid red line represents the assumption that all induced patients had successful vaginal delivery. The solid blue line represents the nomogram.

The outcome of decision curve analysis (DCA) for the nomogram in the training set was presented in Figure 4a. According to the DCA, using the nomogram to determine whether induction should be pursued resulted in a greater net benefit compared with treating all patients or none at all in the training set, given threshold probabilities ranging from 20% to 95%. In other words, the nomogram demonstrated its ability as a potentially useful tool to support clinical decision-making.

### 3.3 Validation of the nomogram predicting vaginal delivery on labor induction

The validation set confirmed the adequacy of the nomogram, demonstrating discrimination with an AUC of 0.760 (95%CI 0.669–0.850) (Figure 3b). The optimal diagnostic cut-off value was determined to be 0.837 based on the Youden index. Sensitivity and specificity were 84.80% and 56.40%, respectively. Furthermore, the validation set exhibited good calibration with a non-significant *P*-value of 0.586 from the Hosmer-Lemeshow test (Figure 3d). DCA revealed that the nomogram provided a higher net benefit in the validation set when the threshold probabilities exceeded 40% (Figure 4b).

## 4 Discussion

### 4.1 Model interpretation

Based on our findings, the probability of successful vaginal delivery in full-term FGR following labor induction was 73.57%. A nomogram model was developed using independent factors including maternal age, multiparity, use of Cook’s double balloon, Bishop score following cervical ripening (all independent predictors with *P* < 0.050), and oligohydramnios (a borderline significant factor with *P* = 0.061 that contributes to clinical utility). This model quantifies and visually represents the outcomes of logistic regression, facilitating the estimation of independent variable values and subsequently predicting the probability of vaginal delivery in individuals with FGR. The nomogram model distinguishes itself from previous clinical models by virtue of its heightened intuitiveness and practicality. Clinicians can use this model to assess the projected rate of vaginal delivery among patients with FGR who elect for labor induction. This empowers patients to select the appropriate approach for terminating the pregnancy.

Our study demonstrated that Cook’s double balloon was associated with a significantly higher rate of successful vaginal delivery (aOR = 2.13) compared with dinoprostone in patients with FGR undergoing induction. This finding is consistent with four recent retrospective and randomized controlled studies (6, 8, 11, 12), but contrasts with a 2020 meta-analysis by Familiari et al. (13) that reported no significant difference in vaginal delivery rates between mechanical and pharmacologic induction methods for FGR. Unlike dinoprostone, Cook’s double balloon exerts a mechanical, gradual stretching force on the cervix (14). This mimics the natural cervical ripening process in spontaneous labor, avoiding the abrupt uterine tachysystole that is common with dinoprostone (15). For FGR fetuses, who are at increased risk of intrapartum hypoxia, the reduced incidence of uterine overstimulation (12.3% vs. 28.7% in our cohort) translates to fewer fetal heart rate abnormalities and fewer unplanned cesarean sections for non-reassuring fetal status. Dinoprostone can cross the placenta and induce fetal ductus arteriosus constriction, a potentially harmful effect in FGR fetuses with compromised placental perfusion (16). Cook’s double balloon, as a mechanical device, has no systemic effects, making it a safer option for FGR pregnancies—an advantage that likely contributes to higher vaginal delivery rates by reducing iatrogenic intervention. However, the efficacy of induction is contingent on the approach used, and it is imperative to consider individual patient characteristics as supplementary elements that impact the outcome of induction. Previous studies (17, 18) documented that variables such as BMI, multiparity, absence of uterine scarring and advanced maternal age could influence the likelihood of vaginal delivery in the induction of FGR pregnancies. Our investigation confirmed that maternal age was associated with a negative effect on the process of labor induction, indicating that advanced age was correlated with a decreased probability of vaginal delivery. A previous study (19) reported a decline in endogenous estrogen levels, estrogen receptors, and estrogen responsiveness as maternal age rises. Additionally, the decreased contractility of uterine smooth muscle reduces the efficacy of labor induction. The beneficial influence of parity on labor induction is clearly evident. Multiparous women exhibit greater compliance in their delivery canal, resulting in easier cervical dilation compared with primiparous women. Prior research (8, 9) has demonstrated that no preeclampsia and a lower pre-gestational BMI enhance the likelihood of successful induction of labor in FGR pregnancies. In contrast, our study did not find any significant correlation between pre-eclampsia, BMI, and the efficacy of labor induction which is consistent with the results reported by Batinelli et al. (20). Most prior studies (21, 22) included unselected term pregnancies, where elevated BMI is linked to cervical dystocia and reduced myometrial contractility. In our FGR cohort, however, induction was performed only when the fetus was deemed mature (37–39 weeks) and the cervix was managed with standardized ripening protocols (12-h device retention, stepwise oxytocin titration). This standardized approach may have mitigated the negative impact of BMI on labor progression—for example, the prolonged cervical ripening period (12 h) allowed even obese patients with unfavorable cervices to achieve adequate dilation before oxytocin initiation, offsetting the usual BMI-related delays.

It has been observed that a low Bishop score before labor induction is associated with a decreased likelihood of vaginal delivery in FGR (23). If the Bishop score is < 2, the rate of vaginal delivery is only 52% (24). However, Daykan et al. (25) have found that a high Bishop score on admission was not correlated with cervical ripening. We also observed no statistically significant disparity in the cervical Bishop score before labor induction. This lack of distinction may be attributed to the inclusion of cases with an immature cervix (2.85 ± 0.86). However, the cervical Bishop score exhibited a noteworthy positive predictive value subsequent to the use of a cervical ripening device. A higher score following labor induction was associated with an increased likelihood of vaginal delivery. Similarly, Lee DS et al. (26) reported that a favorable Bishop score subsequent to cervical ripening was correlated with a decreased rate of CS delivery, even after adjusting for parity and Bishop score at admission. Hence, the selection of an efficacious approach to facilitate cervical maturation assumes paramount importance. Although subjective errors cannot be entirely avoided, the Bishop score retains its status as the principal instrument for appraising cervical status before labor induction and gauging alterations in cervical maturity subsequent to labor induction. To mitigate error, it is imperative for the same practitioner to evaluate cervical maturity before and after labor induction. Additionally, the incorporation of vaginal ultrasound can improve predictive performance by assessing cervical status (27, 28).

### 4.2 Discrepancies between training and validation set performance

Notable differences were observed in the optimal Youden index cut-off values (training set: 0.652; validation set: 0.837) and the balance of sensitivity/specificity between the training and validation sets. The training set exhibited a more balanced performance (sensitivity: 68.30%, specificity: 86.70%), while the validation set showed higher sensitivity (84.80%) but lower specificity (56.40%). These discrepancies are attributable to several factors inherent to predictive model development and validation.

First, the smaller sample size of the validation set (*n* = 127) compared to the training set (*n* = 380) reduces statistical power to capture the full distribution of predicted probabilities, leading to a higher optimal cut-off that prioritizes true positives over true negatives (29). Second, the imbalance in sensitivity and specificity reflects a threshold-driven trade-off: the higher cut-off in the validation set minimizes false negatives but increases false positives, which is clinically acceptable given the ability to monitor patients closely during induction (9).

These discrepancies highlight the importance of contextualizing model performance metrics. The training set’s cut-off (0.652) offers a balanced approach for general use, while the validation set’s higher cut-off (0.837) may be preferred in clinical scenarios where confirming successful induction is prioritized. Future external validation with larger, multi-center cohorts will help refine the optimal threshold and reduce the impact of sample size limitations. Clinicians should also integrate the model’s predicted probabilities with individual patient characteristics and preferences, rather than relying solely on a single cut-off value, to optimize decision-making.

### 4.3 Model calibration and limitations

Calibration of the nomogram was evaluated using the Hosmer-Lemeshow test and calibration curves. The Hosmer-Lemeshow test yielded non-significant *P*-values in both the training set (*P* = 0.991) and validation set (*P* = 0.586), indicating no statistically significant difference between predicted and observed outcomes. However, it is important to acknowledge the limitations of the Hosmer-Lemeshow test in large samples. To address this limitation, we complemented the test with calibration curves (Figures 3c,d), which provide a continuous, visual assessment of calibration. These complementary methods confirm the nomogram’s reliable calibration, mitigating the limitations of the Hosmer-Lemeshow test alone.

The present study developed a prognostic nomogram model for labor induction in pregnancies with FGR. This research provides a valuable resource for enhancing clinical guidelines and facilitating medical decision-making. The research possessed notable strengths, including a largest cohort size of 507 induced patients and a comprehensive assessment of various demographic and clinical factors on FGR pregnancy induction. However, Several limitations of this study warrant consideration. First, the choice of induction method was based on patient preference rather than random allocation, introducing potential selection bias. Patient preferences may be associated with unmeasured factors (e.g., anxiety levels, prior birth experiences, or implicit clinician recommendations) that could confound the association between induction method and vaginal delivery success. Although our model adjusted for key clinical variables, residual confounding may persist. Second, this was a single-center retrospective cohort study, which may limit the generalizability of our findings and increase the risk of selection bias. Third, the exclusion of patients with scarred uterus and lack of Doppler data may restrict the model’s applicability to specific subgroups. Hence, a well-designed, prospective, randomized controlled trial will be advocated in future investigations. The study was conducted at a single center and the external validation of the nomogram’s accuracy has not been performed. Additional investigations are required to assess the predictive accuracy in diverse geographical areas.

## 5 Conclusion

The nomogram model developed in this study may potentially promote labor induction using Cook’s double balloon for most pregnancies with FGR. It is imperative to engage in thorough consultations with patients who are anticipated to have a increased likelihood of vaginal delivery according to our model. This is essential in order to evaluate the potential hazards and advantages of induction and establish the most appropriate course of treatment. In these particular cases, we have the chance to economize both time and resources by abstaining from labor induction and the potential complications associated with an intrapartum CS. The nomogram model demonstrated superior utility in clinical settings compared with traditional logistic regression models due to its straightforwardness, intuitive nature, and practical applicability.

## Data Availability

The raw data supporting the conclusions of this article will be made available by the authors, without undue reservation.
